# Identification of Reference Genes for Quantitative Real-Time PCR in Date Palm (*Phoenix dactylifera* L.) Subjected to Drought and Salinity

**DOI:** 10.1371/journal.pone.0166216

**Published:** 2016-11-08

**Authors:** Himanshu V. Patankar, Dekoum V. M. Assaha, Rashid Al-Yahyai, Ramanjulu Sunkar, Mahmoud W. Yaish

**Affiliations:** 1 Department of Biology, College of Science, Sultan Qaboos University, Muscat, Oman; 2 Department of Crop Science, College of Agriculture and Marine Sciences, Sultan Qaboos University, Muscat, Oman; 3 Department of Biochemistry and Molecular Biology, Oklahoma State University, Stillwater, Oklahoma, United States of America; Universidade de Lisboa Instituto Superior de Agronomia, PORTUGAL

## Abstract

Date palm is an important crop plant in the arid and semi-arid regions supporting human population in the Middle East and North Africa. These areas have been largely affected by drought and salinity due to insufficient rainfall and improper irrigation practices. Date palm is a relatively salt- and drought-tolerant plant and more recently efforts have been directed to identifying genes and pathways that confer stress tolerance in this species. Quantitative real-time PCR (qPCR) is a promising technique for the analysis of stress-induced differential gene expression, which involves the use of stable reference genes for normalizing gene expression. In an attempt to find the best reference genes for date palm’s drought and salinity research, we evaluated the stability of 12 most commonly used reference genes using the geNorm, NormFinder, BestKeeper statistical algorithms and the comparative ΔC_T_ method. The comprehensive results revealed that *HEAT SHOCK PROTEIN* (*HSP*), *UBIQUITIN* (*UBQ*) and YTH domain-containing family protein (*YT521*) were stable in drought-stressed leaves whereas *GLYCERALDEHYDE-3-PHOSPHATE DEHYDROGENASE* (*GAPDH*), *ACTIN* and *TUBULIN* were stable in drought-stressed roots. On the other hand, *SMALL SUBUNIT RIBOSOMAL RNA* (*25S*), *YT521* and 18S ribosomal RNA (*18S*); and *UBQ*, *ACTIN* and *ELONGATION FACTOR 1-ALPHA* (*eEF1a*) were stable in leaves and roots, respectively, under salt stress. The stability of these reference genes was verified by using the abiotic stress-responsive *CYTOSOLIC Cu/Zn SUPEROXIDE DISMUTASE* (*Cyt-Cu/Zn SOD*), an *ABA RECEPTOR*, and a *PROLINE TRANSPORTER 2* (*PRO*) genes. A combination of top 2 or 3 stable reference genes were found to be suitable for normalization of the target gene expression and will facilitate gene expression analysis studies aimed at identifying functional genes associated with drought and salinity tolerance in date palm.

## Introduction

Date palm (*Phoenix dactylifera* L.) is one of the most important socio-economic plants in arid and semi-arid regions, where fresh water is very limited. It is of great spiritual and cultural significance to the people in these areas [1]. In Oman, for example date palms are primary fruit crops comprising about 50% of total agricultural and 80% of total fruit production [2]. Because the availability of fresh water is very limited in this region, the growth and development of date palm is frequently affected by drought stress, which together with salinity, adversely affects plant productivity [3]. Owing to this problem, the number of date palm trees and their production have significantly decreased in recent years [4].

Soil salinization is one of the major abiotic stresses affecting plant growth and productivity worldwide [5]. The main reason for increasing soil salinity is over exploitation of ground water resources near coastal areas, which often leads to sea water mixing with groundwater [6]. Also, irrigation with saline water and lack of rainfall contribute to soil salinity [7]. A number of adaptive mechanisms to salinity and drought have been identified in plants [8,9,10,11,12] and some of the mechanisms are common between these two stresses [13]. These common strategies include upregulation of dehydration-response element-binding proteins [14,15], enhanced water uptake and hydraulic conductivity (aquaporins) [16,17], accumulation of osmolytes (such as sugars, polyols, proline and glycine betaine) and late embryogenesis abundant protein (dehydrins) [18] as well as enhanced antioxidant systems [19].

Profiling stress-induced differential gene expression can yield valuable information on adaptive mechanisms in date palm [20,21]. Quantitative real time PCR (qPCR), which is a commonly used technique to study mRNA abundances, has become a powerful tool for quantifying gene expression. However, for accurate quantification of gene expression, proper reference genes for normalization must be used.

Housekeeping genes are important for basic maintenance of the cell and have a relatively stable expression both under normal and stress conditions as well as developmental stages of the plant, and different tissues within the plant. A number of housekeeping genes such as ß-*ACTIN*, *GLYCERALDEHYDE-3-PHOSPHATE DEHYDROGENASE* (*GAPDH*), 18S *RIBOSOMAL RNA* (*18S*), 25S *RIBOSOMAL RNA* (*25S*), *UBIQUITIN* (*UBQ*), *ELONGATION FACTOR 1-A* (*eEF1α*) and *TUBULIN* have been used as reference genes for normalization of target gene expression in different plant species [22,23,24]. However, wide variation in expression of housekeeping genes exists under stress conditions[25]. Therefore, selection of more stable reference genes is important to correctly measure the abundances of differentially expressed genes. Consequently, before analyzing the expression of target genes under a given stress for a plant of interest it is imperative to first identify stably expressed reference genes. Although the date palm genome has been sequenced and significant portion of the genome has been annotated [26,27,28], most of the genes remain functionally uncharacterized. Furthermore, despite being widely cultivated in stress-prone regions, the molecular basis of stress tolerance is poorly investigated in date palm. As a prelude to functionally characterize stress-associated genes, it is important to establish the stable reference genes for the plant. The present study was therefore undertaken to identify stable reference genes for date palm both under salinity and drought.

## Materials and Methods

### Plant material and growth conditions

Date palm seeds (cv. *Khalas*) were thoroughly washed and surface sterilized with 70% ethanol and incubated in moist sterilized vermiculite at 37°C for 10 days for germination. The germinated seeds were then transferred to 2-L pots containing peat moss and perlite (1:2, v/v). The pots were maintained in a growth chamber with 30°C, 350 μmol.m^-2^.s^-1^ light intensity and a 16 h/8 h light/dark cycle. The plants were monitored and watered to field capacity as required [29].

### Drought and salt stress treatments

To impose treatments, 8-week-old plants were separated into three groups, i.e., control, drought and salinity. Drought was imposed by withholding water for 2 weeks and salt stress by irrigating pots with 300 mM NaCl every 3 days for 2 weeks. The control plants were irrigated with distilled water as required. The electrical conductivity (EC), moisture content and temperature of the soil were monitored regularly using an Em50 Data logger (Decagon Devices, WA, USA).

### Total RNA isolation

Root samples of control, salinity- and drought-stressed plants were crushed to fine powder in pre-chilled mortar, using liquid nitrogen. Total RNA from date palm root was extracted using MRIP extraction buffer as described previously [30]. In brief, MRIP extraction buffer was added to powdered samples, vortexed thoroughly and incubated at room temperature for 5 min. Then, one fifth volume of chloroform was added, mixed thoroughly, incubated at room temperature for 5 min, and centrifuged at 12,000 *g* for 10 min at 4°C. The supernatant was transferred to a fresh tube and an equal volume of chilled isopropanol was added, followed by a 10 min incubation on ice. The precipitated RNA was then pelleted by centrifugation at 12,000 *g* for 10 min at 4°C, washed with cold 70% ethanol and air-dried for 10 min and was re-suspended in 50 μL of nuclease-free water.

Total RNA isolation from leaves was performed as follows. Leaves were pulverized to a fine powder in pre-chilled mortar using liquid nitrogen and 0.5 g powdered sample was transferred to 1.5 mL tubes, to which 500 μL of cold extraction buffer [100 mM Tris-HCl pH 8.0, 0.5% NP-40, 50 mM EDTA, 5% β-mercaptoethanol, 1% PVP] [31] was added and mixed vigorously. After incubating for 10 min at room temperature, phenol-chloroform (5:1, pH 4.5) was added, mixed and the tubes centrifuged at 12,000 *g* for 10 min at 4°C. The supernatant was again extracted with 1/5^th^ volume of chloroform, to which 400 μL of chilled isopropanol and 100 μL of 5 M NaCl solution were added and incubated on ice for 10 min. RNA was later pelleted by centrifuging at 12,000 *g* for 10 min at 4°C, and re-suspended in 50 μL of nuclease-free water.

The extracted RNA from leaf and root tissues was treated with RNase free DNase I (Qiagen, USA), using the RNeasy Plant Mini kit (Qiagen, USA) following the manufacturer’ instructions. The quantity and quality of the RNA was determined spectrophotometrically, using Nanodrop 2000 (Thermo Scientific) and by 1% TAE agarose gel electrophoresis.

### Selection of reference genes and primer designing

Twelve most commonly used reference genes were selected based on gene expression studies in different plant species [22,23,24] (Table 1). The genes from rice were selected and their homologs were searched from the date palm database using BLAST (https://blast.ncbi.nlm.nih.gov/Blast.cgi). Primers were designed using the Primer Express 3.0.1 software (Applied Biosystem, USA), with the following default parameters: optimum primer length 20 bases, minimum length 9 bases and maximum length 40 bases; maximum melting temperature 60°C and minimum 58°C; maximum amplicon length 150 bp and minimum 50 bp; maximum GC content 80% and minimum 30%. The amplification efficiency (E) of the primers were determined from standard curve of serially diluted cDNA using the formula E (%) = (10^−1/slope^—1) × 100. All primers showed E values more than 90%.

**Table 1:** The reference and target genes and their oligonucleotide sequences used for qPCR.

Gene Name	Accession Number	Gene Description	Primer Sequence (5’-3’)		Amplicon length (bp)	Tm	Amplification efficiency	R^2^
			Forward	Reverse				
*18S*	AY012354.1	18S small subunit ribosomal RNA	CGAACCACTGCGAAAGCAT	CCCCCAACTTTCGTTCTTGA	61	72	102.2	0.994
*25S*	XR_602918.1	25S small subunit ribosomal RNA	CCACCGTCCTGCTGTCTTAATC	CGCGCCAACCCAGATC	56	77	100.2	0.998
*ACTIN*	XM_008778129	Actin	TCAATGTGCCTGCCATGTATGT	GCGGCCGCTAGCATAGAG	62	76	100.3	0.997
*eEF1A*	XM_008792592.1	elongation factor 1-alpha-like	GATCCCTTCCTACACTCGAATCC	TCCTTTCCCATTGGTATTTGCT	65	72	102.1	0.992
*EF1*	XM_008790841.1	elongation factor 1-alpha	AATCCAACGGCACGAAGAGA	CAGGAGGGTCGAAGCTTTCA	60	69	99.7	0.983
*GAPDH*	XM_008801419.1	NADP-dependent glyceraldehyde-3-phosphate dehydrogenase	TTTGGACCAGTCTTGCCAGTAA	TGCAGTGATGGATACCTTCTTCA	61	73	106.6	0.990
*HSP*	XM_008812921.1	heat shock protein 81-1-like	TCAAGCTTGGCATCCATGAG	ACCTAAGCAATTCGGCCAACT	60	76	94.0	0.999
*TBP-1*	XM_008810020.1	26S protease regulatory subunit 6A homolog	ACCCCAACTCGTTCAGATGTTT	GCTGAAAGGCATCACGAACAA	62	73	92.4	0.996
*TUBULIN*	XM_008795356	Tubulin beta	GCAAGGAGGCCGAGAACTG	CCTCCCAACGAATGGCATAC	61	76	106.8	0.996
*U6*		Ubiquitin 6	CGATAAAATTGGAACGATACAGA	ATTTGGACCATTCTCGTTTGT		75	96.6	0.994
*UBQ*	XM_008811850.1	ubiquitin-40S ribosomal protein S27a	CCGACACCATCGACAACGT	GCGGGATGCCCTCCTT	54	76	92.6	0.997
*YT521*	XM_008781239.1	YTH domain-containing family protein 2-like	GCAGCTGGTGATGCCTTGA	CATTCGCAATAGCTCGCTTCT	61	76	93.3	0.992
*Cyt-Cu/Zn SOD*	XM_008813474.1	superoxide dismutase [Cu-Zn]-like	AAGCCTCTCTGGCCTCGAA	CACCGAGGGCATGAACATG	56	76	95.9	0.996
*ABA RECEPTOR*	XM_008805920.2	abscisic acid receptor PYL4-like	ACGGTGGGCAGACTCGTAAT	GATCCAGCTTGCAAAAAAAGAAG	61	75	110.6	0.999
*PROLINE TRANSPORTER 2*	XM_008814663.2	proline transporter 2	CCTGGCATTGGTTGAATGTTG	TGGCAGCAGCCAATGCT	54	75	107.9	0.990

### Reverse transcription and Quantitative Real-time PCR

Total RNA (500 ng) was converted into cDNA, using SuperScript^TM^ IV First-Strand Synthesis System kit (Invitrogen, USA) according to the manufacturer’s instructions. The diluted (10 fold) cDNA was used for qPCR in a CFX96 Touch^TM^ Real-Time PCR Detection System (BioRad, USA). For qPCR reactions, 5 μL Fast SYBR® Green Master Mix (Applied Biosystem, USA), 0.1 μL of each primer (10 pmole), 2 μL of diluted cDNA and 2.8 μL of nuclease-free water was used. The reaction conditions used were as follows; 95°C for 20 sec followed by 40 cycles of 95°C for 3 sec, and 60°C for 30 sec. The qPCR was performed using three biological replicates and two technical replicates following MIQE guideline [32].

### Gene expression analysis

The stability of the reference genes was verified using the computer-based statistical programs geNorm [33], Normfinder [34], and BestKeeper, [35] and the comparative ΔC_T_ method [36]. The geNORM is a statistical algorithm which determines the gene stability measure (M) of all genes under investigation, based on the geometric averaging of multiple control genes and mean pairwise variation of a gene from all other control genes in a given sample. The geNORM operates on the principle that the expression ratio of two ideal internal control genes is identical in all samples, regardless of the experimental condition and cell-type. NormFinder is a mathematical model, which also evaluates the expression stability of candidate reference genes. It estimates the stability value of all tested genes based on intra- and inter-group variation. The genes with less stability values are considered to be the most stable, whereas those with highest stability values are ranked as the least stable. BestKeeper is an Excel-based tool that estimates inter-gene relations of possible reference gene pairs by performing numerous pairwise correlation analyses using raw Cq values of each gene. All genes are included in the calculation of the BestKeeper index, which is used to rank the best reference genes since the stable reference genes show strong correlation with the BestKeeper index. Based on the above mentioned programs, the overall stability ranking of the reference genes was estimated using RefFinder [37] by assigning an appropriate weight to an individual gene and calculating the geometric mean of the weights.

### Validation of reference genes

In order to verify the effectiveness of the most stable reference genes for normalizing target gene expression data, three stress-responsive genes Cyt-Cu/Zn superoxide dismutase (*Cyt-Cu/Zn SOD*), abscisic acid receptor (*ABA*), and proline transporter 2 (*PRO*) were selected and the 2^-ΔΔCT^ [38] method was applied to calculate their expressions. All gene expression data were analyzed by one-way ANOVA using the IBM SPSS statistical package version 21 (IBM Corp. Armonk NY USA). Test of significance was performed using the Tukey’s test at α = 0.05.

## Results

### Expression levels of reference genes

**Fig 1:**
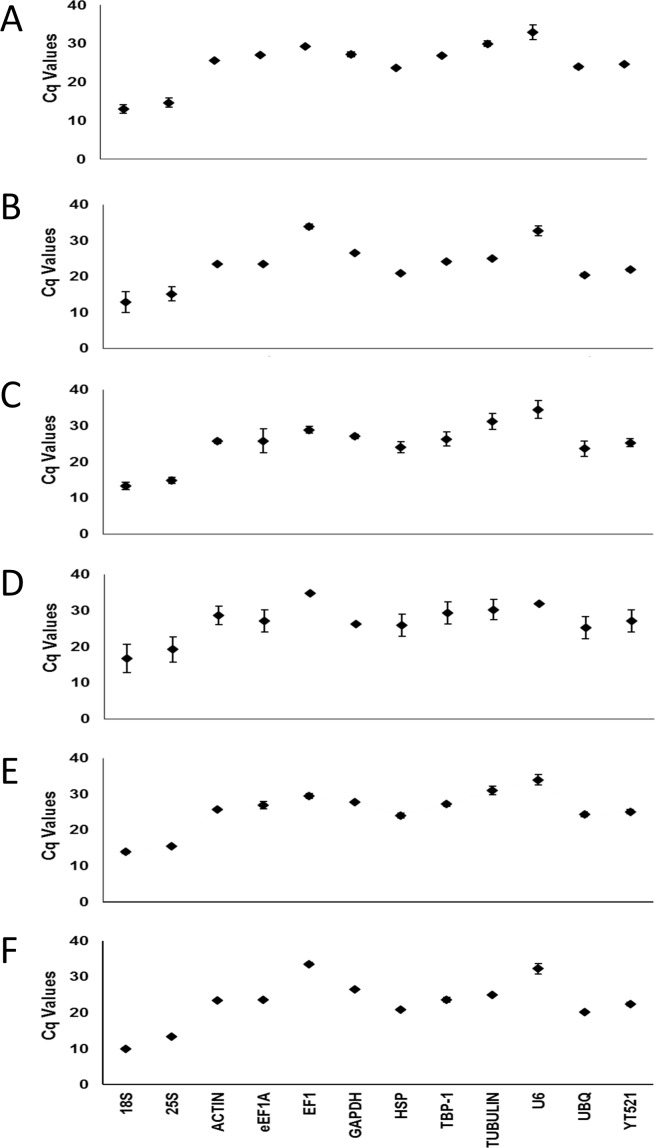
Variation in Cq values for the selected reference genes in date palm leaves and roots under drought and salinity. A) Cq for drought-stressed leaves, B) Cq for drought-stressed roots, C) Cq for salinity-stressed leaves, D) Cq for salinity-stressed roots, E) control leaves, and, F) control roots. Dots on the graph represent median Cq values and the bars are standard deviations.

The median Cq values for the drought treated leaves and roots ranged from 13.07 (*18S*) to 32.98 (*U6*), and 12.86 (*18S*) to 33.82 (*EF1*), respectively. Under salinity, the median Cq values for leaves and roots ranged from 13.31 (*18S*) to 34.57 (*U6*), and 16.72 (*18S*) to 34.81 (*EF1*), respectively. Whereas under control conditions, the median Cq values ranged from 13.89 (*18S*) to 33.97 (*U6*) and 10.4 (*18S*) to 33.58 (*EF1*) for leaves and roots, respectively (Fig 1 and S1 Table).

### Identification of stable reference genes

#### geNorm

**Fig 2:**
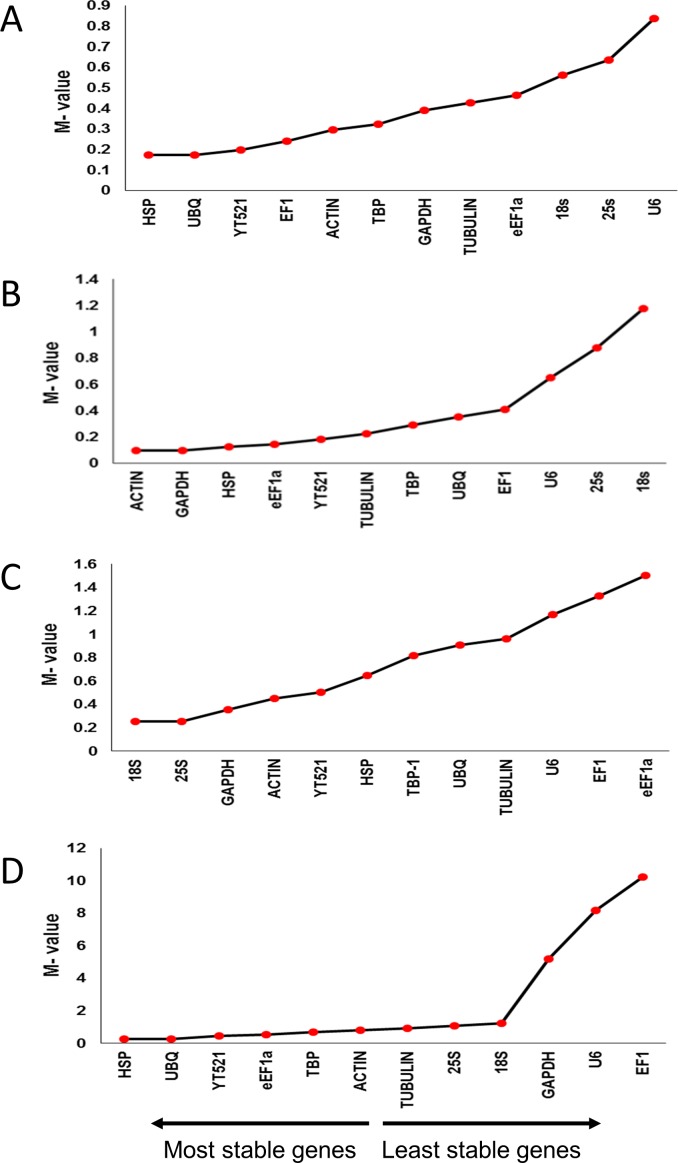
Expression stability analysis of candidate reference genes in drought- and salinity-stressed date palm leaves and roots. A) drought-stressed leaves, B) drought-stressed roots, C) salinity-stressed leaves, and, D) salinity-stressed roots. The gene expression stability graph is based on expression stability values (M-values) obtained with the geNorm algorithm. As per this, lower the M-value, higher the stability of the gene. The arrow direction indicates the order of most stable and least stable reference genes.

The gene expression stability measure (M-value) of the 12 selected reference genes was analyzed using the geNorm algorithm and the rank of each gene was determined. The M-values ranged from 0.174 (*HSP*, *UBQ*) to 0.838 (*U6*) for drought-stressed leaves, and 0.095 (*ACTIN* and *GAPDH*) to 1.176 (*18S*) for drought-stressed roots. The M-values for salinity-treated leaves and roots ranged from 0.253 (*18S*, and *25S*) to 1.503 (*eEF1a*), and 0.276 (*UBQ*, and *HSP*) to 10.210 (*EF1*), respectively (Fig 2, S2, S3, S4 and S5 Tables). According to geNorm the lower the M-value, the higher the stability of the gene, and *vice versa*. The threshold M-value for a stable gene is ≤ 0.5. As per this algorithm, the top stable reference genes for leaves were *HSP* and *YT521* under drought, and *18S* and *25S* under salinity, while for roots, *ACTIN* and *GAPDH* under drought, and *HSP* and *UBQ*, under salinity.

#### NormFinder

**Fig 3:**
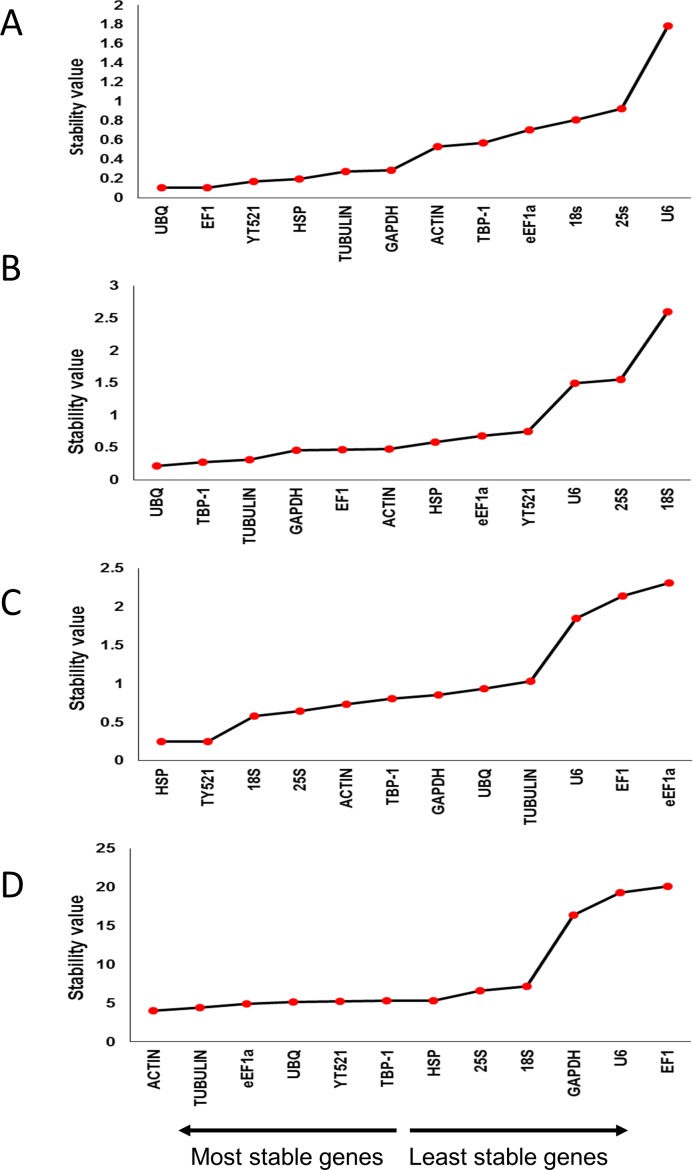
Expression stability analysis of candidate reference genes in drought- and salinity- stressed date palm leaves and roots. A) drought-stressed leaves, B) drought-stressed roots, C) salinity-stressed leaves, and, D) salinity-stressed roots. The gene expression stability graph is based on stability values obtained from the NormFinder algorithm. The lower the stability value, the higher the stability of the gene. The arrow direction indicates the order of most stable and least stable reference genes.

NormFinder is a statistical algorithm, which is used to identify the most stable genes among a set of candidate genes. It ranks the genes according to their expression stability in a given set and experimental conditions. The genes with low stability values are considered to be the most stable, while those with high stability values are less stable. Stability values for leaves under drought and salinity stress ranged from 0.103 (*UBQ*) to 1.788 (*U6*) and 0.245 (*HSP*) to 2.306 (*eEF1a*), respectively. For the roots, the range of stability values under drought and salinity were 0.218 (*UBQ*) to 2.601 (*18S*), and 4.026 (*ACTIN*) to 20.137 (*EF1*), respectively (Fig 3, S2, S3, S4 and S5 Tables).

#### BestKeeper

**Fig 4:**
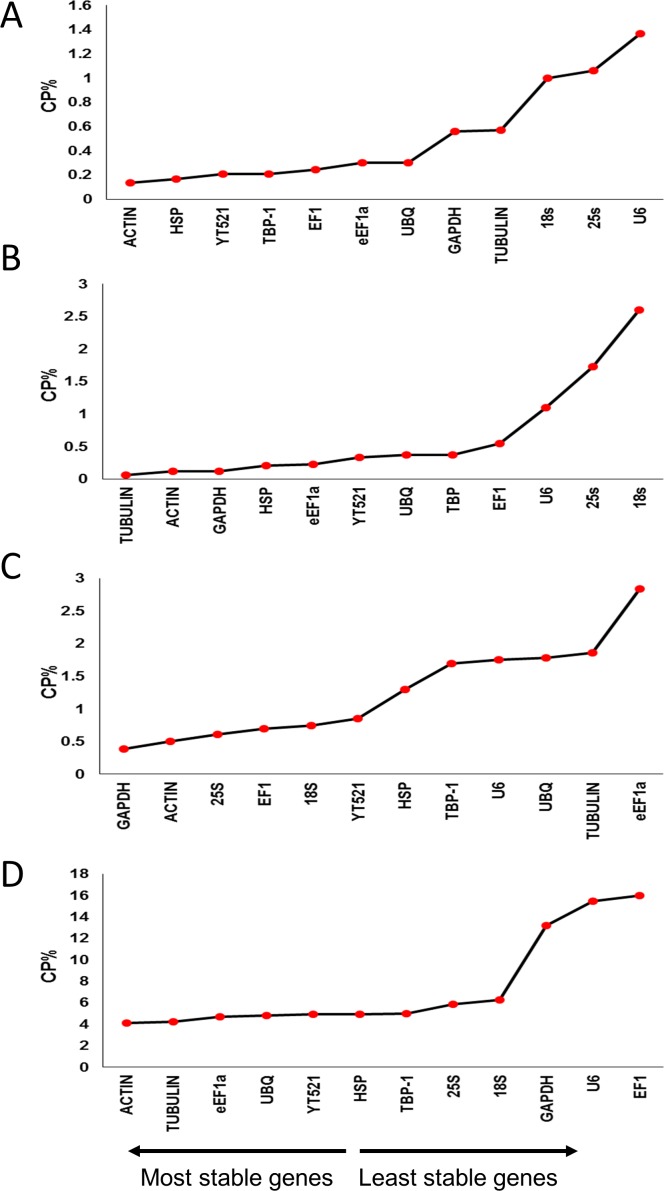
Expression stability analysis of candidate reference genes in drought- and salinity-stressed date palm leaves and roots. A) drought-stressed leaves, B) drought-stressed roots, C) salinity-stressed leaves, and, D) salinity-stressed roots. The gene expression stability graph is based on coefficient of variation (CV) and standard deviation (SD). The lower the CV, the higher the stability of the gene. The arrow direction indicates the order of the most stable and least stable reference genes.

The BestKeeper program was used for the determination of standard deviation (SD) and coefficient of variation (CV) of Cq values, with lower SD and CV representing higher stability. The program uses a statistical method to determine the most stable genes, and compares the differentially expressed target genes under stress conditions on the basis of percentage of crossing points (CP). This analysis suggested that the most stable reference genes for drought-stressed leaves were *ACTIN* and *TUBULIN*, whereas for drought-stressed roots, *ACTIN* and *HSP* were most stable. Under salinity, *GAPDH* and *ACTIN* were most stable in the leaves, while *TUBULIN* and *ACTIN* were most stable in roots (Fig 4, S2, S3, S4 and S5 Tables).

#### Comparative ΔC_T_

**Fig 5:**
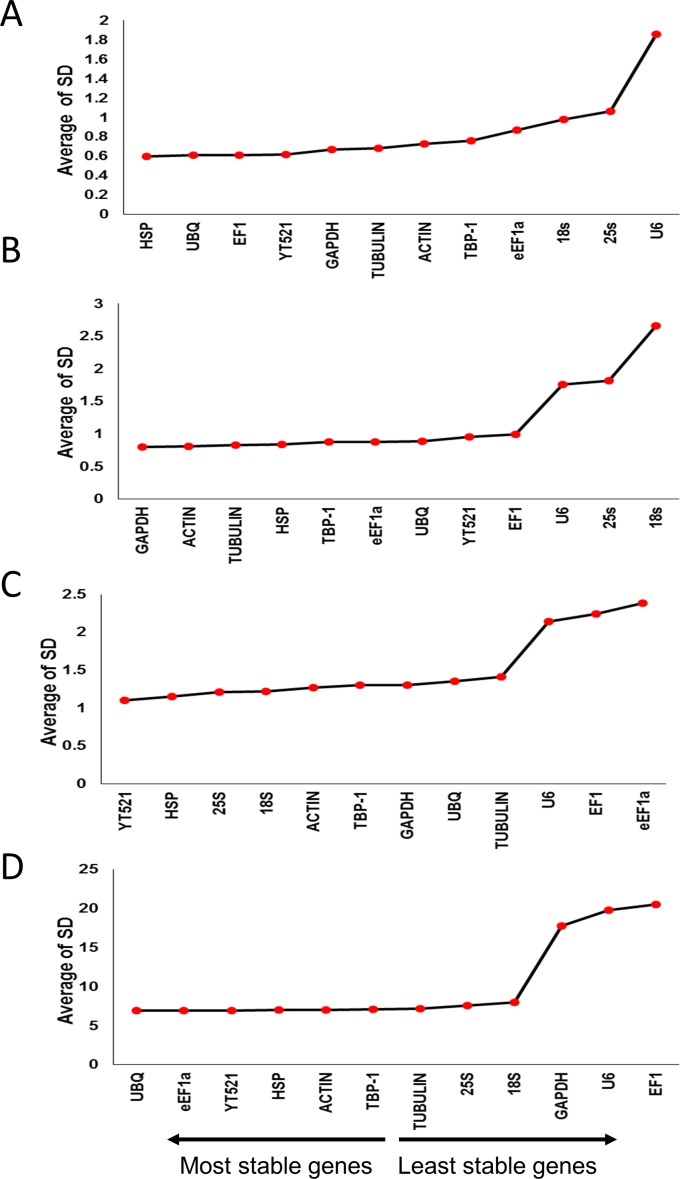
Expression stability analysis of candidate reference genes in drought- and salinity-stressed date palm leaves and roots. A) drought-stressed leaves, B) drought-stressed roots, C) salinity-stressed leaves, and, D) salinity-stressed roots. The gene expression stability graph is based on average standard deviation of variability in Cq values, calculated with the ΔC_T_ method.

The ΔC_T_ method was used to compare the expression of reference genes within each sample and ranks them based on the average of their standard deviation (SD). The results for the ranked values are shown in Fig 5, S2, S3, S4 and S5 Tables. *HSP* and *GAPDH* were found to be the most stable genes in drought-treated leaves and roots, respectively, whereas *YT521* and *UBQ* were the most stable in salinity-treated leaves and roots, respectively.

#### RefFinder (Comprehensive ranking)

**Fig 6:**
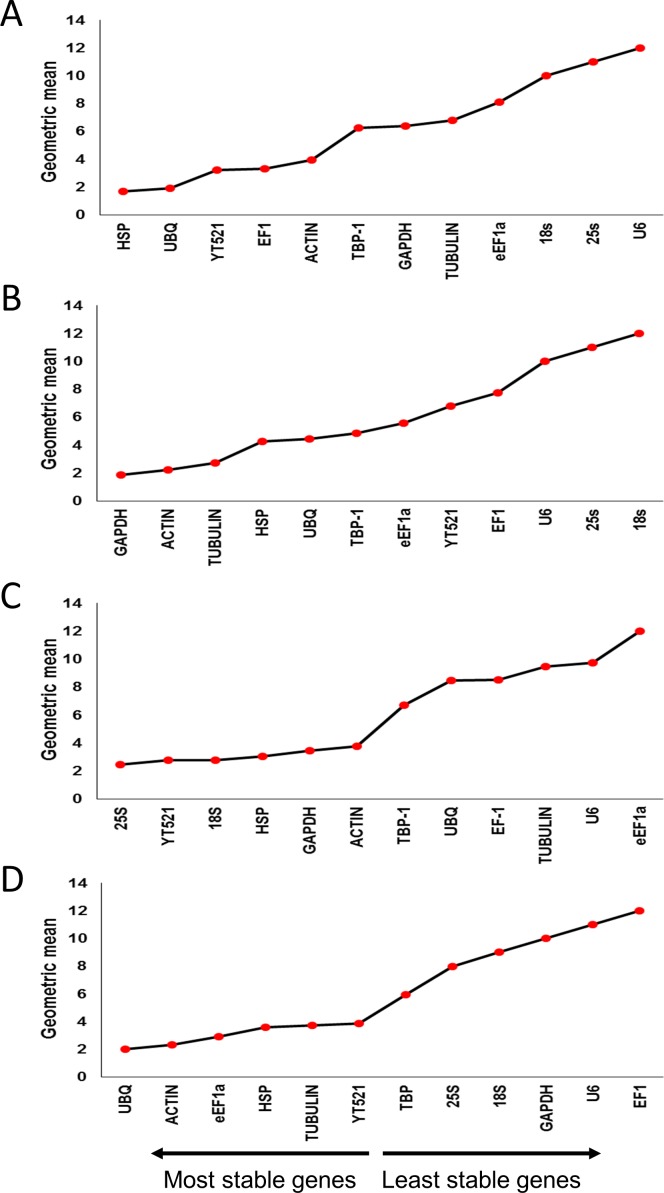
Expression stability analysis of candidate reference genes in drought- and salinity-stressed date palm leaves and roots. A) drought-stressed leaves, B) drought-stressed roots, C) salinity-stressed leaves, and, D) salinity-stressed roots. The gene expression stability graph is based on the geometric mean calculated by RefFinder based on the comprehensive results from the four algorithms. The lower the geometric mean, the higher the stability of the gene.

RefFinder was used to obtain the comprehensive rankings of candidate reference genes by integrating the commonly used analytical programs (geNorm, NormFinder, BestKeeper and ΔC_T_ method). The comprehensive results calculated by RefFinder showed that *HSP* and *UBQ* are most stable in drought-stressed leaves, whereas *GAPDH* and *ACTIN* were most stable in roots. Under salt stress, *25S* and *YT521* in leaves and *UBQ* and *ACTIN* in roots, were most stable (Fig 6, S2, S3, S4 and S5 Tables). The candidate reference genes were analyzed using geNorm, NormFinder, BestKeeper programs individually and the results shared high consistency with the presented results obtained from RefFinder.

### Effect of stable reference genes for relative quantification

**Fig 7:**
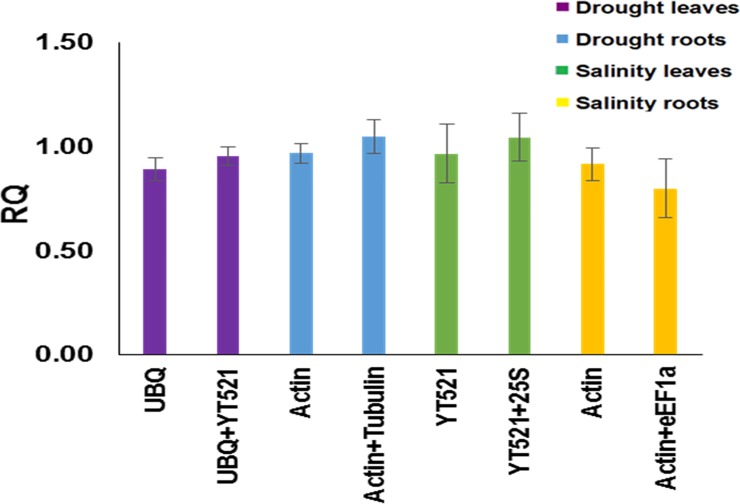
The effect of single reference gene and two reference genes on normalization of most stable gene in each of the four samples (*HSP* for drought-stressed leaves, *GAPDH* for drought-stressed roots, *25S* for salinity-stressed leaves, and, *UBQ* for salinity-stressed roots). Bars represent mean gene relative quantity (RQ) ± SE (n = 3).

The most stable gene under different conditions in leaf and root tissues ranked first in Fig 6 was considered as a target gene and the genes ranking second and third (Fig 6) were considered as reference genes for normalization of gene expression using the 2^-ΔΔCT^ method [38]. Assuming that the target and the reference genes are expressed stably and constantly, the target gene is expected to have a relative quantity value of 1 for a specific tissue sample under a specific condition. The analysis showed that the target gene was stably expressed in all samples, although with slightly lower stable expression in salinity-treated roots (Fig 7).

### Validation of the stability of reference genes

**Fig 8:**
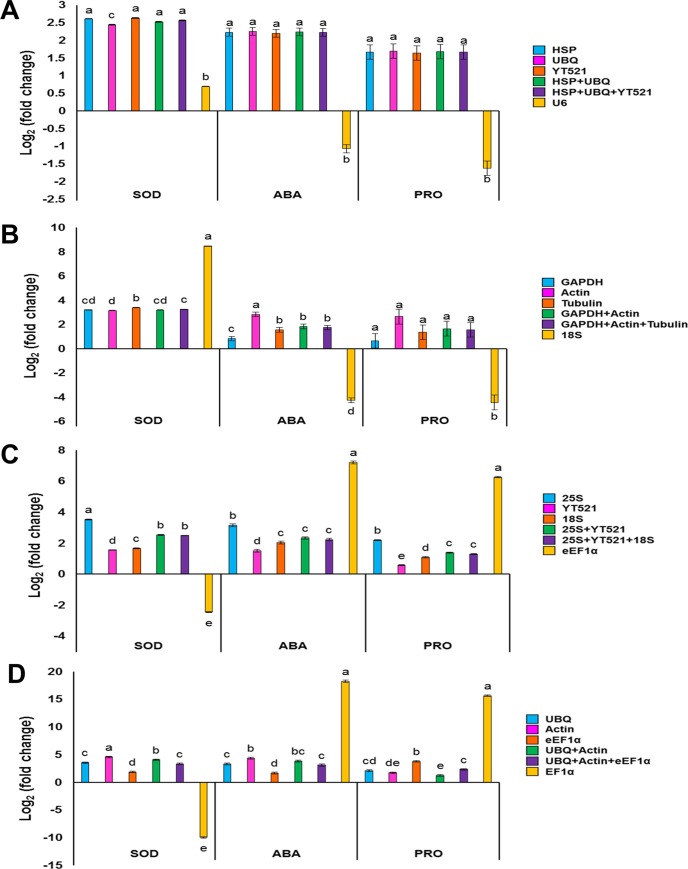
The effect of the most stable and the least stable reference genes on the expression level (fold change) of *Cyt-Cu/ZnSOD* (SOD), abscisic acid receptor (ABA), and proline transporter 2 (PRO) obtained using the qPCR in drought-stressed leaves (A), drought-stressed roots (B), salinity-stressed leaves (C) and salinity-stressed roots (D). Bars represent mean log _2_ fold change ± SE (n = 3), lower case letters indicates significant difference at *P* ≤ 0.05.

The *Cyt-Cu/Zn SOD*, ABA receptor, and proline transporter 2 genes, which are induced under both salinity and drought were used as target genes to verify the expression stability of the reference genes. These 3 genes are known to participate in ROS scavenging (dismutation of O_2_^-^ by SOD), limiting water loss by regulating stomatal aperture (ABA signaling) and osmotic adjustment to sustain cell turgidity and cellular metabolism (proline synthesis and accumulation). The top three most stable reference genes for each sample obtained from the RefFinder algorithm were used to normalize the expression of these 3 genes (*Cyt-Cu/ZnSOD*, ABA receptor, and proline transporter 2) (Fig 8). Using the top three reference genes independently to normalize the target gene expression revealed some differences in the expression patterns, whereas normalization using a combination of the top two and three reference genes showed stable expression patterns in the different tissues. The results showed a 3–4 fold increase in expression of the *Cyt-Cu/Zn SOD*, 2–3 fold increase in ABA receptor gene and approximately 2 fold increase in proline transporter gene in the drought- and salinity-treated date palm leaf and root tissues. Normalization of gene expression using the most stable genes individually showed variation in the expression pattern except in the case of ABA receptor and proline transporter 2 genes in drought-stressed leaves, where a uniform pattern of gene expression was observed. On the contrary, using combination of the top stable reference genes revealed stable expression under drought and salinity stress in leaves and roots, with the exception of proline transporter 2 gene in salinity-stressed roots, where a combination of the top three genes (*UBQ*, *ACTIN*, *eEF1α*) showed relatively stable expression than the combination of the top two gene (*UBQ*, *ACTIN*). However, using the least stable reference genes (*U6*, *18S*, *eEF1α*, *EF1α*) to normalize the expression value in the same analysis showed profound variations in the gene expression levels in leaves and roots in response to salinity and drought.

## Discussion

Understanding the adaptive mechanisms of plants under drought and salinity is a major focal point in agronomic research. Gene expression analysis can unravel such adaptive mechanisms. However gene expression data is only meaningful when it is normalized with an appropriate stable reference gene. qPCR is a decisive technique, which is routinely used in the rapid and accurate quantification of gene expression. However, the efficiency of the qPCR results depends mainly on the reference gene used for normalizing target gene expression. This warrants a need to mine for stable reference genes under different environmental conditions. Ideally, reference genes should have the same level of expression irrespective of environmental conditions. However, none of the reference genes satisfies this criteria and the analyses of different plant species suggest the use of specific reference gene under a particular condition, as the expression of reference genes can vary under different conditions [39]. Some authors have even suggested using more than one reference gene for the normalization, following differential expression of reference genes in different tissues of a plant species [40]. Therefore, it is necessary to evaluate the stability of different reference genes for accurate target gene expression analysis in different plant tissues. Thus far, suitable reference gene(s) for date palm’s drought and salinity research has not been reported. In the present study we selected 12 different reference genes to assess and validate their stability in date palm. The study yielded 4 candidate genes that are stably expressed under specific stress conditions, and in different tissues, which can be suitably used as reference genes to normalize gene expression in date palm.

In this study, geNorm, Normfinder, Bestkeeper, and ΔCq_,_ the most widely used algorithms for assessing gene stability were used to calculate the expression stability of the reference genes. According to geNorm, the lower the M-value, the higher the stability of the gene, and *vice versa* [33]. The ranking results based on gene stability values obtained from the NormFinder algorithm were highly similar to those from geNorm. The results obtained for the stable reference genes in date palm were quite variable from those of other plants [23,24,41] suggesting high interspecific variation in stability of reference genes. The use of RefFinder to comprehensively find the most stable reference genes based on the above 4 algorithms produced *HSP* and *GAPDH* as the most stable in drought-stressed leaves and roots, respectively, while *25S* and *UBQ* were most stable in salinity-treated leaves and roots, respectively.

Previous studies reported that the *UBQ* showed stable expression in *Arabidopsis* and *Populus* [42,43], *GAPDH* in sugarcane [41,44] and *EF1a* in potato [41]. Studies in *Capsicum annuum* reported *GAPDH* and *TUBULIN* as the most suitable reference genes for normalizing gene expression [45]. In rice, *UBQ5* and *eEF-1α* were the most stable reference genes across different tissues and developmental stages and hence recommended either single or their combination for normalizing gene expression [40]. Contrarily, our study yielded no such gene(s) for normalizing gene expression across two tissues and stress conditions. However, our study has identified tissue-specific and stress-specific genes, which are suitable for normalizing target gene expression in date palm.

Under drought, *HSP* in leaves and *GAPDH* in roots were found to be the most stable, whereas under salinity, *25S* in leaves and *UBQ* in roots were found to be most stable genes. *ACTIN* was ranked as the second most stable reference gene in roots both under salinity and drought. *YT512* was among the third most stable reference gene in leaves under salinity and drought. The *18S* and *25S* genes were most stable in salinity-treated leaves, but were least stable in drought-stressed leaves. *GAPDH* was one of the stable genes in drought-stressed roots, whereas this was not the case in salinity-treated roots. However, *TBP-1* was found to be moderately stable in both leaves and roots under both stresses.

The suitability of reference genes for normalizing target gene expression ultimately depends on their ability to produce invariable expression of the target genes. This assessment has previously been used to confirm the suitability of various reference genes, singly or in combination, in different plant species, tissues and conditions [46,47,48,49,50]. In the current study, 3 genes (*Cyt-Cu/Zn SOD*, ABA receptor and proline transporter 2) that are inducible by drought and salinity [29,51,52], were selected to validate the suitability of the most stable reference genes obtained from RefFinder (Fig 6). High expression levels of three target genes were observed, when normalized using the top three stable reference genes (Fig 8). Usage of the top stable reference genes individually for normalization showed modest variation in the expression, whereas target gene normalization using the combination of top two and three genes showed uniform and stable expression, hence suitable for gene expression analysis. Normalization of target gene expression using the least stable reference genes produced large variation in the pattern of gene expression (Fig 8) clearly indicating the unsuitability of these genes for normalization of gene expression. The gene expression of the target genes normalized using the most stable and the least stable reference genes showed biased expression, which was significantly different at *P* ≤ 0.05. The expression of *Cyt-Cu/Zn SOD* gene was higher in roots than in leaves under both drought and salinity stress. ABA receptor gene was expressed higher in salinity-stressed roots than the other samples. Similarly proline transporter 2 gene was expressed at higher levels in salinity-stressed roots. Hence the combination of *HSP*, *UBQ*, and *YT521*, and *GAPDH*, *ACTIN*, and *TUBULIN* was best suited for normalization of gene expression in drought-stressed date palm leaves and roots, respectively. Similarly, for salinity-stressed date palm leaves and roots, combining *25S*, *YT521*, and *18S* and *UBQ*, *ACTIN*, and *eEF1α* yielded more consistent stable expressions, respectively. Thus, this study revealed the variation in the pattern of expression of different reference genes in leaves and roots in response to drought and salinity.

Selection of stable reference genes for gene expression analysis in date palm root was a major challenge due to the fact that isolating RNA of high quality and quantity was not easy. This could explain why gene expression studies on date palm roots were very limited. In the present study we were able to identify stable reference gene in roots under different stress conditions. Previous studies on root transcriptional analysis of date palm under short term salinity shock [20] used *ACTIN* and *TUBULIN* as reference genes for normalization of gene expression. The present study has clearly shown that while ACTIN is stable and hence suitable as reference gene, *TUBULIN* is only moderately stable in salinity-stressed date palm roots and thus not very suitable for normalizing gene expression under salinity stress.

In conclusion, the present study identified *HSP* and *UBQ* in leaves, or *GAPDH* and *ACTIN* in roots under drought, whereas *YT512* and *25S* in leaves or *UBQ* and *ACTIN* in roots were the most stable reference genes under salinity. Amongst the identified stably expressed reference genes in date palm, the top 2 or 3 genes depending on the tissue and environmental conditions for normalizing target gene expression were highly recommended.

## Supporting Information

S1 Table Cq values with +/-SD of all the samples and the reference genes. (DOCX)

S2 Table Stability values of reference genes for date palm leaves under drought, according to different algorithms. (DOCX)

S3 Table Stability values of reference genes for date palm root under drought, according to different algorithms. (DOCX)

S4 Table Stability values of reference genes for date palm leaves under salinity, according to different algorithms. (DOCX)

S5 Table Stability values of reference genes for date palm roots under salinity, according to different algorithms. (DOCX)

## References

[1] Chao CT, Krueger RR. The date palm (*Phoenix dactylifera* L.): Overview of biology, uses, and cultivation. HortScience. 2007; 42: 1077–1082.

[2] Al-Yahyai R, Khan MM. Date Palm Status and Perspective in Oman In: Al-Khayri JM, Jain SM, Johnson DV, editors. Date palm genetic resources and utilization. Netherlands: Springer Science and Business Media; 2015 pp. 207–240. 10.1007/978-94-017-9707-8_6

[3] Yaish MW, Kumar PP. Salt Tolerance Research in Date Palm Tree (*Phoenix dactylifera* L.), Past, Present and Future Perspectives. Front Plant Sci. 2015; 6 10.3389/fpls.2015.00348 26042137 PMC4434913

[4] El-Juhany LI. Degradation of date palm trees and date production in Arab countries: causes and potential rehabilitation. Aust J Basic Appl Sci. 2010; 4: 3998–4010.

[5] Munns R, Tester M. Mechanisms of salinity tolerance. Annu Rev Plant Biol. 2008; 59: 651–681. 10.1146/annurev.arplant.59.032607.092911 18444910

[6] Stanger G. Coastal salinization: A case history from Oman. Agric Water Manag. 1985; 9: 269–286.

[7] Malash N, Flowers T, Ragab R. Effect of irrigation methods, management and salinity of irrigation water on tomato yield, soil moisture and salinity distribution. Irrigation Sci. 2008; 26: 313–323.

[8] Deinlein U, Stephan AB, Horie T, Luo W, Xu G, Schroeder JI. Plant salt-tolerance mechanisms. Trends Plant Sci. 2014; 19: 371–379. 10.1016/j.tplants.2014.02.001 24630845 PMC4041829

[9] Maathuis FJ, Ahmad I, Patishtan J. Regulation of Na^+^ fluxes in plants. Front Plant Sci. 2014; 5: 467 10.3389/fpls.2014.00467 25278946 PMC4165222

[10] Zhu J-K. Plant salt tolerance. Trends Plant Sci. 2001; 6: 66–71. 11173290 10.1016/s1360-1385(00)01838-0

[11] Wang W, Vinocur B, Altman A. Plant responses to drought, salinity and extreme temperatures: towards genetic engineering for stress tolerance. Planta. 2003; 218: 1–14. 10.1007/s00425-003-1105-5 14513379

[12] Farooq M, Wahid A, Kobayashi N, Fujita D, Basra S. Plant drought stress: effects, mechanisms and management Sustainable Agriculture: Springer; 2009 pp. 153–188.

[13] Zhu J-K. Salt and drought stress signal transduction in plants. Annu Rev Plant Biol. 2002; 53: 247 10.1146/annurev.arplant.53.091401.143329 12221975 PMC3128348

[14] Zhao TJ, Sun S, Liu Y, Liu JM, Liu Q, Yan YB, et al Regulating the drought-responsive element (DRE)-mediated signaling pathway by synergic functions of trans-active and trans-inactive DRE binding factors in Brassica napus. J Biol Chem. 2006; 281: 10752–10759. 10.1074/jbc.M510535200 16497677

[15] Bhatnagar-Mathur P, Sharma KK, Devi MJ, Serraj R, Yamaguchi-Shinozaki K, Vadez V. Evaluation of transgenic groundnut lines under water limited conditions. INA. 2004; 24: 33–35.

[16] Javot H, Maurel C. The role of aquaporins in root water uptake. Ann Bot. 2002; 90: 301–313. 10.1093/aob/mcf199 12234142 PMC4240399

[17] Maurel C, Javot H, Lauvergeat V, Gerbeau P, Tournaire C, Santoni V, et al Molecular physiology of aquaporins in plants. Int Rev Cytol. 2002; 215: 105–148. 11952226 10.1016/s0074-7696(02)15007-8

[18] Close TJ. Dehydrins: a commonalty in the response of plants to dehydration and low temperature. Physiol Plant. 1997; 100: 291–296.

[19] Bartels D, Sunkar R. Drought and salt tolerance in plants. Crit Rev Plant Sci. 2005; 24: 23–58.

[20] Radwan O, Arro J, Keller C, Korban SS. RNA-Seq Transcriptome Analysis in Date Palm Suggests Multi-Dimensional Responses to Salinity Stress. Trop Plant Biol. 2015; 8: 74–86.

[21] Yaish MW, Sunkar R, Zheng Y, Ji B, Al-Yahyai R, Farooq SA. A genome-wide identification of the miRNAome in response to salinity stress in date palm (*Phoenix dactylifera* L.). Front Plant Sci. 2015; 6 10.3389/fpls.2015.00946 26594218 PMC4633500

[22] Sinha P, Saxena RK, Singh VK, Varshney RK. Selection and validation of housekeeping genes as reference for gene expression studies in pigeonpea (*Cajanus cajan*) under heat and salt stress conditions. Front Plant Sci. 2015; 6: 1071 10.3389/fpls.2015.01071 27242803 PMC4865767

[23] Sinha P, Singh VK, Suryanarayana V, Krishnamurthy L, Saxena RK, Varshney RK. Evaluation and validation of housekeeping genes as reference for gene expression studies in Pigeonpea (*Cajanus cajan*) under drought stress conditions. PloS one. 2015; 10: e0122847 10.1371/journal.pone.0122847 25849964 PMC4388706

[24] Wang X, Ma X, Huang L, Zhang X. Identification of the Valid Reference Genes for Quantitative RT-PCR in Annual Ryegrass (*Lolium multiflorum*) under Salt Stress. Molecules. 2015; 20: 4833–4847. 10.3390/molecules20034833 25786166 PMC6272566

[25] Liu C, Wu G, Huang X, Liu S, Cong B. Validation of housekeeping genes for gene expression studies in an ice alga Chlamydomonas during freezing acclimation. Extremophiles. 2012; 16: 419–425. 10.1007/s00792-012-0441-4 22527038

[26] Al-Mssallem IS, Hu S, Zhang X, Lin Q, Liu W, Tan J, et al Genome sequence of the date palm *Phoenix dactylifera* L. Nat Commun. 2013; 4. 10.1038/ncomms3274 PMC3741641 23917264

[27] Al-Dous EK, George B, Al-Mahmoud ME, Al-Jaber MY, Wang H, Salameh YM, et al De novo genome sequencing and comparative genomics of date palm (*Phoenix dactylifera*). Nat Biotechnol. 2011; 29: 521–527. 10.1038/nbt.1860 21623354

[28] Hazzouri KM, Flowers JM, Visser HJ, Khierallah HS, Rosas U, Pham GM, et al Whole genome re-sequencing of date palms yields insights into diversification of a fruit tree crop. Nat Commun. 2015; 6. 10.1038/ncomms9824 PMC4667612 26549859

[29] Yaish M. Proline accumulation is a general response to abiotic stress in the date palm tree (*Phoenix dactylifera* L.). Genet Mol Res. 2015; 14: 9943–9950. 10.4238/2015.august.19.30 26345930

[30] Xiao Y, Yang Y, Cao H, Fan H, Ma Z, Lei X, et al Efficient isolation of high quality RNA from tropical palms for RNA-seq analysis. Plant Omics. 2012; 5: 584.

[31] Saïdi MN, Gargouri-Bouzid R, Rayanni M, Drira N. Optimization of RNA isolation from brittle leaf disease affected date palm leaves and construction of a subtractive cDNA library. Mol Biotechnol. 2009; 41: 63–68. 10.1007/s12033-008-9104-1 18815905

[32] Bustin SA, Benes V, Garson JA, Hellemans J, Huggett J, Kubista M, et al The MIQE guidelines: minimum information for publication of quantitative real-time PCR experiments. Clin Chem. 2009; 55: 611–622. 10.1373/clinchem.2008.112797 19246619

[33] Vandesompele J, De Preter K, Pattyn F, Poppe B, Van Roy N, De Paepe A, et al Accurate normalization of real-time quantitative RT-PCR data by geometric averaging of multiple internal control genes. Genome Biol. 2002; 3: 1–12. 10.1186/gb-2002-3-7-research0034 PMC126239 12184808

[34] Andersen CL, Jensen JL, Ørntoft TF. Normalization of real-time quantitative reverse transcription-PCR data: a model-based variance estimation approach to identify genes suited for normalization, applied to bladder and colon cancer data sets. Cancer Res. 2004; 64: 5245–5250. 10.1158/0008-5472.CAN-04-0496 15289330

[35] Pfaffl MW, Tichopad A, Prgomet C, Neuvians TP. Determination of stable housekeeping genes, differentially regulated target genes and sample integrity: BestKeeper–Excel-based tool using pair-wise correlations. Biotechnol Lett. 2004; 26: 509–515. 15127793 10.1023/b:bile.0000019559.84305.47

[36] Silver N, Best S, Jiang J, Thein SL. Selection of housekeeping genes for gene expression studies in human reticulocytes using real-time PCR. BMC Mol Biol. 2006; 7: 1. doi: 10.1186/1471-2199-9-28 17026756 PMC1609175

[37] Xie F, Xiao P, Chen D, Xu L, Zhang B. miRDeepFinder: a miRNA analysis tool for deep sequencing of plant small RNAs. Plant Mol Biol. 2012; 80: 75–84. 10.1007/s11103-012-9885-2 22290409

[38] Livak KJ, Schmittgen TD. Analysis of relative gene expression data using real-time quantitative PCR and the 2^− ΔΔCT^ method. Methods. 2001; 25: 402–408. 10.1006/meth.2001.1262 11846609

[39] Thellin O, Zorzi W, Lakaye B, De Borman B, Coumans B, Hennen G, et al Housekeeping genes as internal standards: use and limits. J Biotechnol. 1999; 75: 291–295. 10617337 10.1016/s0168-1656(99)00163-7

[40] Jain M, Nijhawan A, Tyagi AK, Khurana JP. Validation of housekeeping genes as internal control for studying gene expression in rice by quantitative real-time PCR. Biochem Biophys Res Commun. 2006; 345: 646–651. 10.1016/j.bbrc.2006.04.140 16690022

[41] Nicot N, Hausman J-F, Hoffmann L, Evers D. Housekeeping gene selection for real-time RT-PCR normalization in potato during biotic and abiotic stress. J Exp Bot. 2005; 56: 2907–2914. 10.1093/jxb/eri285 16188960

[42] Czechowski T, Stitt M, Altmann T, Udvardi MK, Scheible WR. Genome-wide identification and testing of superior reference genes for transcript normalization in Arabidopsis. Plant Physiol. 2005; 139: 5–17. 10.1104/pp.105.063743 16166256 PMC1203353

[43] Brunner AM, Yakovlev IA, Strauss SH. Validating internal controls for quantitative plant gene expression studies. BMC Plant Biol. 2004; 4: 1. doi: 10.1186/1471-2229-4-1 15317655 PMC515301

[44] Iskandar HM, Simpson RS, Casu RE, Bonnett GD, Maclean DJ, Manners JM. Comparison of reference genes for quantitative real-time polymerase chain reaction analysis of gene expression in sugarcane. Plant Mol Biol Report. 2004; 22: 325–337.

[45] Wan H, Yuan W, Ruan M, Ye Q, Wang R, Li Z, et al Identification of reference genes for reverse transcription quantitative real-time PCR normalization in pepper (*Capsicum annuum* L.). Biochem Biophys Res Commun. 2011; 416: 24–30. 10.1016/j.bbrc.2011.10.105 22086175

[46] Saha P, Blumwald E. Assessing reference genes for accurate transcript normalization using quantitative real-time PCR in pearl millet [*Pennisetum glaucum* (L.) R. Br.]. PloS one. 2014; 9: e106308 10.1371/journal.pone.0106308 25170776 PMC4149553

[47] Yang Z, Chen Y, Hu B, Tan Z, Huang B. Identification and validation of reference genes for quantification of target gene expression with quantitative real-time PCR for tall fescue under four abiotic stresses. PloS one. 2015; 10: e0119569 10.1371/journal.pone.0119569 25786207 PMC4364999

[48] Amil-Ruiz F, Garrido-Gala J, Blanco-Portales R, Folta KM, Muñoz-Blanco J, Caballero JL. Identification and validation of reference genes for transcript normalization in strawberry (*Fragaria*× *ananassa*) defense responses. PloS one. 2013; 8: e70603 10.1371/journal.pone.0070603 23940602 PMC3734262

[49] Reddy DS, Bhatnagar-Mathur P, Cindhuri KS, Sharma KK. Evaluation and validation of reference genes for normalization of quantitative real-time PCR based gene expression studies in peanut. PloS one. 2013; 8: e78555 10.1371/journal.pone.0078555 24167633 PMC3805511

[50] Gimeno J, Eattock N, Van Deynze A, Blumwald E. Selection and validation of reference genes for gene expression analysis in switchgrass (*Panicum virgatum*) using quantitative real-time RT-PCR. PloS one. 2014; 9: e91474 10.1371/journal.pone.0091474 24621568 PMC3951385

[51] Golldack D, Li C, Mohan H, Probst N. Tolerance to drought and salt stress in plants: unraveling the signaling networks. Front Plant Sci. 2014;5 10.3389/fpls.2014.00151 24795738 PMC4001066

[52] Sah SK, Reddy KR, Li J. Abscisic acid and abiotic stress tolerance in crop plants. Front Plant Sci. 2016; 7 10.3389/fpls.2016.00571 27200044 PMC4855980

