# Supplementary material for: Identification of Reference Genes for Quantitative Real-Time PCR in Date Palm (Phoenix dactylifera L.) Subjected to Drought and Salinity
Source: PLoS One. 2016 Nov 8;11(11):e0166216. doi: 10.1371/journal.pone.0166216 (PMC5100987; doi:10.1371/journal.pone.0166216)
Supplement: S1 Table — (DOCX) [file pone.0166216.s001.docx]

**S1 Table**. Cq values with +/-SD of all the samples and the reference genes.

|  | Control Leaves | +/-SD | Control Roots | +/-SD | Drought leaves | +/-SD | Drought roots | +/-SD | Salinity leaves | +/-SD | Salinity roots | +/-SD |
| --- | --- | --- | --- | --- | --- | --- | --- | --- | --- | --- | --- | --- |
| 18S | 14.06 | 0.15 | 10.18 | 0.27 | 12.07 | 0.22 | 15.38 | 0.13 | 12.465 | 1.41 | 10.85 | 0.88 |
| 25S | 15.74 | 0.17 | 13.44 | 0.26 | 13.62 | 0.41 | 16.90 | 0.23 | 14.105 | 1.14 | 13.395 | 0.13 |
| ACTIN | 25.65 | 0.07 | 23.52 | 0.24 | 25.73 | 0.31 | 23.40 | 0.08 | 25.195 | 0.21 | 24.795 | 0.36 |
| eEF1A | 26.73 | 0.23 | 23.68 | 0.33 | 27.33 | 0.14 | 23.28 | 0.16 | 22.815 | 0.63 | 22.975 | 1.11 |
| EF1 | 29.54 | 0.11 | 33.61 | 0.45 | 29.05 | 0.13 | 34.15 | 0.79 | 29.695 | 0.70 | 31.98 | 3.93 |
| GAPDH | 27.84 | 0.10 | 26.74 | 0.35 | 26.72 | 0.07 | 26.66 | 0.14 | 26.585 | 0.70 | 26.41 | 0.45 |
| HSP | 23.86 | 0.18 | 21.05 | 0.32 | 23.66 | 0.21 | 20.79 | 0.21 | 22.525 | 0.63 | 21.59 | 0.69 |
| TBP-1 | 26.84 | 0.31 | 23.74 | 0.62 | 27.04 | 0.17 | 24.18 | 0.19 | 24.51 | 0.72 | 24.385 | 0.54 |
| TUBULIN | 30.71 | 0.33 | 25.01 | 0.06 | 29.56 | 0.13 | 25.11 | 0.07 | 29.1 | 1.03 | 26.905 | 2.07 |
| U6 | 33.27 | 1.57 | 31.84 | 1.50 | 32.70 | 2.50 | 32.67 | 1.45 | 33.09 | 3.90 | 30.895 | 0.80 |
| UBQ | 24.12 | 0.28 | 20.01 | 0.43 | 23.75 | 0.41 | 20.75 | 0.21 | 21.665 | 0.70 | 20.85 | 0.45 |
| YT521 | 24.84 | 0.23 | 22.29 | 0.48 | 24.65 | 0.31 | 21.81 | 0.19 | 24.26 | 0.71 | 22.265 | 0.15 |
