# Supplementary material for: Identification of Reference Genes for Quantitative Real-Time PCR in Date Palm (Phoenix dactylifera L.) Subjected to Drought and Salinity
Source: PLoS One. 2016 Nov 8;11(11):e0166216. doi: 10.1371/journal.pone.0166216 (PMC5100987; doi:10.1371/journal.pone.0166216)
Supplement: S2 Table — (DOCX) [file pone.0166216.s002.docx]

**S2 Table.** Stability values of housekeeping genes for date palm leaves under drought stress conditions, according to different algorithms.

| Rank | RefFinder | | geNorm | | NormFinder | | Comparative ∆CT | | BestKeeper | |
| --- | --- | --- | --- | --- | --- | --- | --- | --- | --- | --- |
|  | Genes | Geomean of  ranking values | Genes | Normalization  Value (M-value) | Gene | Stability value | Genes | Avg of STDEV | Genes | CP(%)+/-SD |
| 1 | HSP | 1.68 | HSP | 0.174 | UBQ | 0.103 | HSP | 0.6 | ACTIN | 0.136 |
| 2 | UBQ | 1.93 | UBQ | 0.174 | EF1 | 0.108 | UBQ | 0.61 | HSP | 0.17 |
| 3 | YT521 | 3.22 | YT521 | 0.197 | YT521 | 0.17 | EF1 | 0.61 | YT521 | 0.209 |
| 4 | EF1 | 3.31 | EF1 | 0.239 | HSP | 0.197 | YT521 | 0.62 | TBP-1 | 0.21 |
| 5 | ACTIN | 3.96 | ACTIN | 0.295 | TUBULIN | 0.275 | GAPDH | 0.67 | EF1 | 0.247 |
| 6 | TBP-1 | 6.26 | TBP | 0.322 | GAPDH | 0.284 | TUBULIN | 0.68 | eEF1a | 0.3 |
| 7 | GAPDH | 6.4 | GAPDH | 0.391 | ACTIN | 0.532 | ACTIN | 0.73 | UBQ | 0.303 |
| 8 | TUBULIN | 6.82 | TUBULIN | 0.426 | TBP-1 | 0.57 | TBP-1 | 0.76 | GAPDH | 0.558 |
| 9 | eEF1a | 8.13 | eEF1a | 0.463 | eEF1a | 0.703 | eEF1a | 0.87 | TUBULIN | 0.573 |
| 10 | 18S | 10 | 18S | 0.561 | 18S | 0.812 | 18S | 0.98 | 18S | 0.998 |
| 11 | 25S | 11 | 25S | 0.635 | 25S | 0.927 | 25S | 1.06 | 25S | 1.063 |
| 12 | U6 | 12 | U6 | 0.838 | U6 | 1.788 | U6 | 1.86 | U6 | 1.365 |
