# Supplementary material for: Identification of Reference Genes for Quantitative Real-Time PCR in Date Palm (Phoenix dactylifera L.) Subjected to Drought and Salinity
Source: PLoS One. 2016 Nov 8;11(11):e0166216. doi: 10.1371/journal.pone.0166216 (PMC5100987; doi:10.1371/journal.pone.0166216)
Supplement: S3 Table — (DOCX) [file pone.0166216.s003.docx]

**S3 Table.** Stability values of housekeeping genes for date palm root under drought stress conditions, according to different algorithms.

| Rank | RefFinder | | geNorm | | NormFinder | | Comparative ∆CT | | BestKeeper | |
| --- | --- | --- | --- | --- | --- | --- | --- | --- | --- | --- |
|  | Genes | Geomean of  ranking values | Genes | Normalization value (M-value) | Gene | Stability value | Genes | Avg of STDEV | Genes | CP(%)+/-SD |
| 1 | GAPDH | 1.86 | ACTIN | 0.095 | UBQ | 0.218 | GAPDH | 0.8 | TUBULIN | 0.063 |
| 2 | ACTIN | 2.21 | GAPDH | 0.095 | TBP-1 | 0.269 | ACTIN | 0.81 | ACTIN | 0.119 |
| 3 | TUBULIN | 2.71 | HSP | 0.126 | TUBULIN | 0.315 | TUBULIN | 0.83 | GAPDH | 0.119 |
| 4 | HSP | 4.28 | eEF1a | 0.144 | GAPDH | 0.458 | HSP | 0.84 | HSP | 0.203 |
| 5 | UBQ | 4.45 | YT521 | 0.181 | EF1 | 0.464 | TBP-1 | 0.88 | eEF1a | 0.231 |
| 6 | TBP-1 | 4.86 | TUBULIN | 0.223 | ACTIN | 0.478 | eEF1a | 0.88 | YT521 | 0.332 |
| 7 | eEF1a | 5.57 | TBP | 0.291 | HSP | 0.582 | UBQ | 0.89 | UBQ | 0.37 |
| 8 | YT521 | 6.82 | UBQ | 0.355 | eEF1a | 0.675 | YT521 | 0.95 | TBP | 0.37 |
| 9 | EF1 | 7.77 | EF1 | 0.411 | YT521 | 0.751 | EF1 | 0.99 | EF1 | 0.545 |
| 10 | U6 | 10 | U6 | 0.651 | U6 | 1.49 | U6 | 1.76 | U6 | 1.1 |
| 11 | 25S | 11 | 25S | 0.88 | 25S | 1.556 | 25S | 1.82 | 25S | 1.728 |
| 12 | 18S | 12 | 18S | 1.176 | 18S | 2.601 | 18S | 2.66 | 18S | 2.6 |
