# Supplementary material for: Identification of Reference Genes for Quantitative Real-Time PCR in Date Palm (Phoenix dactylifera L.) Subjected to Drought and Salinity
Source: PLoS One. 2016 Nov 8;11(11):e0166216. doi: 10.1371/journal.pone.0166216 (PMC5100987; doi:10.1371/journal.pone.0166216)
Supplement: S4 Table — (DOCX) [file pone.0166216.s004.docx]

**S4 Table.** Stability values of housekeeping genes for date palm leaves under salinity stress conditions, according to different algorithms.

| Rank | RefFinder | | geNorm | | NormFinder | | Comparative ∆CT | | BestKeeper | |
| --- | --- | --- | --- | --- | --- | --- | --- | --- | --- | --- |
|  | Genes | Geomean of ranking values | Genes | Normalization value (M-value) | Gene | Stability value | Genes | Avg of STDEV | Genes | CP(%)+/-SD |
| 1 | 25S | 2.45 | 18S | 0.253 | HSP | 0.245 | YT521 | 1.1 | GAPDH | 0.391 |
| 2 | YT521 | 2.78 | 25S | 0.253 | TY521 | 0.248 | HSP | 1.15 | ACTIN | 0.508 |
| 3 | 18S | 2.78 | GAPDH | 0.353 | 18S | 0.578 | 25S | 1.21 | 25S | 0.613 |
| 4 | HSP | 3.03 | ACTIN | 0.452 | 25S | 0.64 | 18S | 1.22 | EF1 | 0.7 |
| 5 | GAPDH | 3.48 | YT521 | 0.502 | ACTIN | 0.732 | ACTIN | 1.27 | 18S | 0.749 |
| 6 | ACTIN | 3.76 | HSP | 0.65 | TBP-1 | 0.808 | TBP-1 | 1.3 | YT521 | 0.855 |
| 7 | TBP-1 | 6.7 | TBP-1 | 0.818 | GAPDH | 0.854 | GAPDH | 1.3 | HSP | 1.298 |
| 8 | UBQ | 8.46 | UBQ | 0.908 | UBQ | 0.937 | UBQ | 1.35 | TBP-1 | 1.695 |
| 9 | EF-1 | 8.54 | TUBULIN | 0.963 | TUBULIN | 1.029 | TUBULIN | 1.41 | U6 | 1.75 |
| 10 | TUBULIN | 9.46 | U6 | 1.168 | U6 | 1.849 | U6 | 2.14 | UBQ | 1.785 |
| 11 | U6 | 9.74 | EF1 | 1.33 | EF1 | 2.139 | EF1 | 2.24 | TUBULIN | 1.86 |
| 12 | eEF1a | 12 | eEF1a | 1.506 | eEF1a | 2.306 | eEF1a | 2.38 | eEF1a | 2.843 |
