# Supplementary material for: Identification of Reference Genes for Quantitative Real-Time PCR in Date Palm (Phoenix dactylifera L.) Subjected to Drought and Salinity
Source: PLoS One. 2016 Nov 8;11(11):e0166216. doi: 10.1371/journal.pone.0166216 (PMC5100987; doi:10.1371/journal.pone.0166216)
Supplement: S5 Table — (DOCX) [file pone.0166216.s005.docx]

**S5 Table.** Stability values of housekeeping genes for date palm roots under salinity stress conditions, according to different algorithms.

| Rank | RefFinder | | geNorm | | NormFinder | | Comparative ∆CT | | BestKeeper | |
| --- | --- | --- | --- | --- | --- | --- | --- | --- | --- | --- |
|  | Genes | Geomean of ranking values | Genes | Normalization value (M-value) | Gene | Stability value | Genes | Avg of STDEV | Genes | CP(%)+/-SD |
| 1 | UBQ | 2 | HSP | 0.273 | ACTIN | 4.026 | UBQ | 6.89 | ACTIN | 4.128 |
| 2 | ACTIN | 2.34 | UBQ | 0.273 | TUBULIN | 4.425 | eEF1a | 6.91 | TUBULIN | 4.25 |
| 3 | eEF1a | 2.91 | YT521 | 0.472 | eEF1a | 4.936 | YT521 | 6.92 | eEF1a | 4.688 |
| 4 | HSP | 3.6 | eEF1a | 0.532 | UBQ | 5.137 | HSP | 6.99 | UBQ | 4.843 |
| 5 | TUBULIN | 3.74 | TBP | 0.669 | YT521 | 5.257 | ACTIN | 7.01 | YT521 | 4.933 |
| 6 | YT521 | 3.87 | ACTIN | 0.796 | TBP-1 | 5.332 | TBP-1 | 7.11 | HSP | 4.96 |
| 7 | TBP | 5.96 | TUBULIN | 0.94 | HSP | 5.34 | TUBULIN | 7.2 | TBP-1 | 4.968 |
| 8 | 25S | 8 | 25S | 1.09 | 25S | 6.643 | 25S | 7.6 | 25S | 5.89 |
| 9 | 18S | 9 | 18S | 1.241 | 18S | 7.19 | 18S | 7.96 | 18S | 6.262 |
| 10 | GAPDH | 10 | GAPDH | 5.198 | GAPDH | 16.405 | GAPDH | 17.78 | GAPDH | 13.205 |
| 11 | U6 | 11 | U6 | 8.165 | U6 | 19.294 | U6 | 19.74 | U6 | 15.448 |
| 12 | EF1 | 12 | EF1 | 10.214 | EF1 | 20.137 | EF1 | 20.46 | EF1 | 15.99 |
